# Supplementary material for: Food and Nutrition in Autistic Adults: Knowledge Gaps and Future Perspectives
Source: Nutrients. 2025 Apr 26;17(9):1456. doi: 10.3390/nu17091456 (PMC12073154; doi:10.3390/nu17091456)
Supplement: Supplementary file 1 [file nutrients-17-01456-s001.zip › nutrients-3579895-supplementary.pdf]

Table S1: **Food selectivity in autistic adults**

Reference	Aim	Study Design & Sample	Methodology for outcomes of interest	Results & Conclusions
**Kuschner *et al.*, 2015**<br>[33]<br>USA	To examine self-reported food selectivity in adolescents and young adults with Autism Spectrum Disorder (ASD) and compare it to typically developing controls.	• Design: Preliminary quantitative study (self-report questionnaires)<br>• Sample:<br>○ Participants included 65 adolescents/young adults with ASD (ages 12-28) and 59 typically developing controls (ages 12-23).<br>○ Participants were matched on age, IQ, and sex ratio.	• Self-ratings of food selectivity were obtained using portions of the Adult/Adolescent Sensory Profile (AASP).<br>• Items analyzed included preferences for familiar foods (food neophobia), dislike for particular food textures, and reactions to strong tastes.<br>• Parent ratings of adaptive behavior skills were collected using the Adaptive Behavior Assessment System-II (ABAS-II).	• Adolescents and young adults with ASD were more likely to be food neophobic compared to typically developing peers.<br>• Individuals with ASD reported disliking textured foods and strong tastes more than controls.<br>• Food neophobia in individuals with ASD was associated with lower parent ratings of daily living skills<br>• There is a need for effective intervention strategies to address food selectivity in older individuals with higher functioning ASD.
**Barbier, 2015**<br>[34]<br>USA	To qualitatively explore the relationship between eating behaviors and autism using a questionnaire and interviews with adults diagnosed with autism spectrum disorder.	• Design: Qualitative study using interviews and the Swedish Eating Assessment for Autism Spectrum Disorders (SWEAA) questionnaire.<br><br>• Sample: Four male participants aged 22-27 , all diagnosed with autism spectrum disorder	• Each of the participants had their mothers present for both the interview and questionnaire to ensure accuracy of the interview and questionnaire completion.<br>• Participants were recruited through a local support group.<br>• Procedure: Interviews conducted, Informed consent obtained, Completion of the SWEAA questionnaire.<br>• Data Collection and Analysis:<br>o Interviews transcribed and analyzed through open and axial coding.<br>o Questionnaires analyzed for consistent findings or discrepancies with interview responses.	• Recognizing Hunger and Satiety: Participants could recognize hunger and satiety but had difficulty articulating these feelings.<br>• Typical Diet and Eating Behaviors: High carbohydrate, high fat diet, low in vegetables; food preferences influenced by family encouragement.<br>• Change in Eating Habits Over Time: Improved variety in diet with age; negative responses to new foods decreased.<br>• Attitude Toward Trying New Foods: Generally neutral or reluctant, influenced by past negative experiences.<br>• Body Image and Weight: No concern for body image; parents concerned about weight gain as participants aged.<br>• Communication methods, environment, and nutrition education influence eating patterns in adults with autism.
**Kinnaird *et al.,* 2019**<br>[29]<br>UK	To explore whether autism impacts eating for some autistic individuals in adulthood and how far this is perceived by these individuals as a problem.	• Design: Exploratory qualitative study<br>• Sample:<br>○ 12 adults with ASD. 38.5+13.9 (range 19-71) years.<br>○ BMI: 2 normal weight; 4 underweight associated to ED; 6 overweight/obese	• Participants were recruited from a previous online study on problematic eating in autism.<br>• Eligible participants were over 18 and self-reported an autism diagnosis.<br>• Semi-structured interviews were conducted via video conferencing, phone, or instant messenger. Interviews focused on eating behaviors and aspects known to be atypical in autism, such as sensory sensitivity and selectivity.<br>• Thematic analysis was used to analyze the transcribed interviews (Autism and eating; Impact; Coping and adapting)	• All participants described a degree of selectivity around their eating and food choices; Participants described avoiding certain foods or seeking out specific foods. Some foods were avoided linked to hypersensitivities to taste, texture, smell, and temperature. Eating similar foods repeatedly was also described<br>• All participants felt that autism influenced their eating to some degree, but most of them felt that can be managed. It also impacts how they behaved in social situations involving eating.<br>• Most participants did not perceive the influence of autism on eating as difficult or problematic; Participants

Reference	Aim	Study Design & Sample	Methodology for outcomes of interest	Results & Conclusions
				generally described becoming more flexible around food as they became older<br>● Traits associated with autism, such as cognitive rigidity and sensory sensitivity, can influence eating behaviors in autistic adults.
Folta *et al.*, 2020<br>[35]<br>USA	To explore the impact of selective eating on key social domains—with family, peers, and in other social situations—of transition-age autistic youth who self-identified as being food selective.	● Design: Qualitative study, semi-structured, in-depth interviews with autistic youth<br>● Sample: Conducted 20 autistic young adults aged 18–23 years.	● Inclusion criteria included being 18–23 years old, diagnosed with ASD, self-identified as a picky eater, and able to participate in interviews.<br>● Interviews were conducted via Zoom and analyzed using descriptive and thematic coding.<br>● Participants completed a set of questionnaires prior to the interview to characterize the study population.	● Participants had a range of food preferences and aversions, often related to sensory issues.<br>● The impact of selective eating diminished with age, and participants developed coping strategies.<br>● Selective eating did not significantly impact social participation due to developed coping strategies.<br>● Participants did not express interest in receiving help for their selective eating.
Pubilsky-Yanofchick *et al.,* 2022<br>[36]<br>USA	To evaluate behavioral treatments to increase the acceptance of novel foods, specifically fruits and vegetables, in an adult with autism spectrum disorder (ASD)	● Design: Intervention study: Combined alternating-treatments and changing-criterion design.<br>● Sample: A 26-year-old male with ASD, who had a long history of food selectivity.	● Sessions were conducted at an employment support center and a campus dining hall.<br>● Response Measurement and Interobserver Agreement:<br>o Frequency data on food acceptance were collected<br>o Interobserver agreement was 94%.<br>● Treatment:<br>o Multiple-stimulus without-replacement (MSWO) preference assessments<br>o Differential reinforcement of alternative (DRA) (DRA + positive and DRA -negative)	● Both DRA+ and DRA- treatments increased acceptance of food<br>● the DRA+ condition was preferred when given a choice.<br>● Treatment effects transferred to novel foods and settings<br>● Food selectivity is also a problem for adults with ASD.<br>● Differential reinforcement can be an effective treatment for food selectivity exhibited by individuals with a long history of restricted food-related repertoires.
Waldron *et al.*, 2022<br>[31]<br>USA	To describe self-care practices of adults on the Autism Spectrum (AS) and explore the self-reported impact of such practices on their health and well-being.	● Design: Exploratory qualitative study.<br>● Sample:<br>o 29 adults with ASD (range 51-79) years<br>o 77% diagnosed by a medical professional.<br>o BMI: 2 normal weight; 4 underweight associated to ED; 6 overweight/obese	• Interviews covered topics such as physical health, mental health, services and supports, employment/retirement, and social relationships.<br>•Data were analyzed using Dedoose software and a constant comparative method.	● The key findings about food selectivity in the study are:<br>o Food Sensory Issues: Many participants reported limited diets due to food sensory issues and unfavorable food textures, such as soft or stringy foods.<br>o Routine and Consistency: Some participants relied on routine and consistency in their eating habits,<br>o Limited Cooking Skills: A significant barrier to good nutrition was the inability to cook or prepare healthy meals.

ASD = Autism Spectrum Disorder

Table S2: **Food sensory processing in autistic adults**

Reference	Aim	Study Design & Sample	Methodology for outcomes of interest	Results & Conclusions
**Tavassoli & Baron-Cohen, 2012** [37] UK	To investigate taste identification accuracy and error types in adults with Autism Spectrum Conditions (ASC) using 'Taste Strips'.	• Design: Comparative study.<br>• Sample:<br>o 23 adults with ASC (21 with Asperger Syndrome, 2 with High-Functioning Autism). Mean age in years 35,8.<br>o 26 control participants with no psychiatric conditions: Mean age in years 25,1.	• Participants were instructed not to eat or drink anything other than water for an hour before the test.<br>• 'Taste Strips' impregnated with sweet, sour, salty, and bitter tastes were used.<br>• Participants identified the taste from a list of four descriptors (sweet, sour, salty, bitter) after each strip was placed on their tongue.	• Adults with ASC had significantly lower taste identification scores overall compared to control participants.<br>• Specifically, lower scores were observed for bitter, sour, and sweet tastes in the ASC group.<br>• No significant difference was found for salty taste identification between the groups.<br>• Error analysis showed that adults with ASC more often misidentified tastes as salty or as no taste.
**Tavassoli & Baron-Cohen, 2012** [38] UK	To investigate olfactory detection thresholds and adaptation to olfactory stimuli in adults with Autism Spectrum Condition (ASC).	• Design: Comparative exploratory study.<br>• Sample:<br>o 38 adults with ASC (Age: 35.9) and 42 control participants (28.8).<br>o A subgroup of 19 participants from each group conducted an adaptation task.	• Olfactory Detection Threshold Task:<br>o Used standardized "Sniffin' Sticks" to measure olfactory detection levels.<br>o Participants were blindfolded and presented with sticks containing different concentrations of n-butanol.<br>• Olfactory Adaptation Task:<br>o After measuring detection thresholds, participants were exposed to the highest concentration of butanol for 30 seconds before measuring detection thresholds again.<br>o The task was repeated with 10-second breaks between trials.	• No significant difference in olfactory detection thresholds between the ASC group and the control group.<br>• Both groups showed similar adaptation to the olfactory stimulus, with no significant differences in detection thresholds before and after adaptation.<br>• The study suggests that olfactory adaptation is normal in adults with ASC, contrasting with previous findings of diminished adaptation in other sensory modalities.
**Mayer, 2017** [39] UK	To examine specific patterns across aspects of autistic traits and sensory	• Design: correlational study<br>• Sample	• Participants completed questionnaires for the Autism Spectrum Quotient (AQ) and Adult/Adolescent Sensory Profile (AASP) online	• The relationship between autistic traits and sensory functioning is similar in NT and ASD adults.<br>• Sensory atypicalities exist throughout the neurotypical population and are not specific to ASD.

Reference	Aim	Study Design & Sample	Methodology for outcomes of interest	Results & Conclusions
	behaviors within both ASD and NT populations.	○ Recruited 580 NT adults and 42 high-functioning ASD adults with a confirmed diagnosis.<br>○ Mean age of 35.07 (SD=12.38).	• The AQ assessed five factors: Social Skills, Attention Switching, Attention to Detail, Communication, and Imagination.<br>• The AASP assessed sensory functioning across six sensory modalities and derived quadrant scores: Low Registration, Sensation Seeking, Sensory Sensitivity, and Sensation Avoiding.	• The broader autism phenotype is evident, with a clear progression of atypical sensory behaviors in line with increasing autistic traits.<br>• Lower sensation seeking behaviors may be characteristic of individuals with increased autistic symptomatology.
Avery *et al.*, 2018<br>[40]<br>USA	To examine the neural correlates of taste reactivity in individuals with autism spectrum disorder (ASD) and compare them with typically developing (TD) controls	• Design: Comparative exploratory study.<br>• Sample:<br>○ 21 males with ASD and 21 TD males, aged 15-29. Age (21 ± 3)<br>○ Participants with ASD met DSM-5 criteria and had IQ scores ≥ 80.<br>○ Exclusion criteria included neurological injury, genetic or medical disorders, prenatal drug exposure, and psychiatric conditions for TD participants.	• Behavioural assessments using the Adolescent/Adult Sensory Profile (AASP) to measure taste reactivity.<br>• Functional magnetic resonance imaging (fMRI) to evaluate hemodynamic responses to sweet (vs. neutral) tastants and food pictures.<br>• Resting-state functional connectivity scans.<br>• Gustatory Mapping task involved delivering tastant solutions during fMRI scanning.<br>• Food Picture task involved viewing pictures of foods and non-food objects during fMRI scanning.	• No significant differences in hemodynamic response to gustatory stimuli between ASD and TD groups.<br>• Positive association between self-reported taste reactivity and response to sweet tastants in the insular cortex and other brain regions in ASD subjects.<br>• Significant interaction between diagnostic group and taste reactivity on tastant response in brain regions associated with ASD pathophysiology, including the bilateral anterior superior temporal sulcus (STS).<br>• Similar interaction observed in resting-state functional connectivity between the anterior STS and dorsal mid-insula.<br>• Heightened taste reactivity in ASD is associated with heightened brain responses to food-related stimuli and atypical functional connectivity of primary gustatory cortex.<br>• These neural differences may predispose individuals with ASD to maladaptive and unhealthy patterns of selective eating behavior.
Brede *et al.*, 2020<br>[41]<br>UK	To better understand how Anorexia Nervosa (AN) develops and persists in autistic individuals from the perspective of autistic women, parents, and healthcare professionals.	• Design: Qualitative research design using semi-structured interviews.<br>• Sample: 15 autistic women (Age 32,6), 13 parents of autistic women (Age 24,75), and 16 healthcare professionals.	• Semi-structured interviews were conducted face-to-face, via Skype, or over the phone.<br>• Thematic Analysis was used to identify patterns of meaning across the data.<br>• Interviews covered experiences of autism, AN, factors underlying the development of AN, and service experiences.<br>• The 10-item Autism-Spectrum Quotient (AQ-10) was used to confirm autism status.<br>• The Eating Disorders Examination Questionnaire Short (EDE-QS) was used to measure current ED psychopathology.	• Sensory sensitivities were a significant factor contributing to restrictive eating behaviors in autistic women.<br>• Sensory overload from noise, touch, and lighting was reported, affecting day-to-day life and eating behaviors.<br>• Food-specific sensory sensitivities, such as aversions to certain textures, tastes, smells, and temperatures, limited the range of foods consumed.<br>• Sensory-related restrictive eating behaviors were present since early childhood and continued to interfere with eating even after recovery from AN.
Singh & Seo, 2022	To achieve a better understanding of how	• **Design: Exploratory study based on interviews**	• Structured interviews with scripted questions were conducted.	• Reactivities to sensory inputs vary among autistic individuals.

Reference	Aim	Study Design & Sample	Methodology for outcomes of interest	Results & Conclusions
[42]<br>USA	atypical eating behaviors might be associated with specific sensory functions and consumption environments through firsthand accounts of autistic individuals.	• Sample: 23 autistic adults' ages ranged from 19 to 55 years, with a mean age of 26 years	• Interviews focused on sensory experiences in general, sensory experiences during eating or drinking, and eating behavior and food preference.<br>• Data were transcribed and analyzed using a general summative data analysis approach.	• High sensitivity to sound cues was commonly reported.<br>• Sensory cues reflecting qualities of sourness, bitterness, or spiciness were considered highly intense and often led to a dislike of food or beverage items with such cues.<br>• Atypical sensitivities in each modality were found to affect food choice and preference.<br>• Environmental factors such as perfume, other people's chatting, loud music, and specific light or utensil conditions were found to influence eating experiences.
Nisticò *et al.,* 2023<br>[43]<br>ITALY	To evaluate the relationship between sensory sensitivity and autistic eating behaviours or Eating Disorders (EDs) symptomatology in adults with Autism Spectrum Disorders (ASDs) without intellectual disabilities.	• Design: Observational exploratory study<br>• Sample: 75 adults with ASDs without intellectual disabilities (aged 36,13)	• Participants completed several self-report questionnaires:<br>o Ritvo Autism Asperger Diagnostic Scale-Revised (RAADS-R)<br>o Sensory Perception Quotient-Short Form 35 item (SPQ-SF35)<br>o Eating Attitude Test (EAT-26)<br>o Swedish Eating Assessment for Autism Spectrum Disorders (SWEAA)	• Hypersensitivity in the vision domain predicted higher levels of both EDs symptoms and autistic eating behaviours.<br>• Hyposensitivity in the taste domain predicted higher levels of EDs symptoms.<br>• Specific subscales of the SWEAA and EAT-26 were significantly associated with sensory sensitivity scores.<br>• Sensory sensitivity, particularly in the vision and taste domains, is associated with dysfunctional eating behaviours in adults with ASDs without intellectual disabilities
Nisticò *et al.,* 2024<br>[44]<br>ITALY	To investigate the prevalence of eating disorder symptomatology and its potential relationship with autistic traits and sensory sensitivity in young adults	• Design: Design: Observational exploratory study<br>• Sample: 259 young adults aged 18-24 years who were referred to a mental health clinic	• Participants completed an online questionnaire including sociodemographic information and four validated self-report questionnaires:<br>o Eating Attitude Test (EAT-26)<br>o Swedish Eating Assessment for Autism Spectrum Disorders (SWEAA)<br>o Autism Quotient (AQ)<br>o Ritvo Autism Asperger Diagnostic Scale-Revised (RAADS-R)<br>o Sensory Perception Quotient—Short Form 35 item (SPQ-SF35)	• 23.55% of participants scored above the cut-off on the EAT-26, indicating a risk for eating disorders.<br>• Associations were found between hypersensitivity in the touch and vision domains and both the EAT-26 and SWEAA scores.<br>• The presence of autistic traits was significantly associated with eating disturbances.<br>• The findings suggest that altered sensory sensitivity and subthreshold autistic traits may play a role in the development of dysfunctional eating behaviors.

ASD = Autism Spectrum Disorder

Table S3: **Clinical, biochemical, and dietary assessment of autistic adults' nutritional status**

Reference	Aim	Study Design & Sample	Methodology for outcomes of interest	Results & Conclusions
**Biochemical assessment**				
**Kočovská *et al*, 2014**<br>[56]<br>Faroe Islands (Denmark)	The aim was to explore vitamin D (25(OH)D3) levels in a population-based study of young adults with ASD in the Faroe Islands and compare them to siblings, parents, and healthy controls.	• Cross-sectional population-based study<br>• First ever entire population study of ASD individuals' vitamin D levels (N = 219):<br>○ 40 individuals with ASD formally diagnosed: 23% females, aged 15-24 years, % ID not reported<br>○ 62 typically developing siblings: 53% females	• Vitamin D status was stablished by the serum levels of 25(OH)D_3 (nmol/L) and the following reference cut-offs:<br>○ Severe deficiency: <25<br>○ Deficiency: $\geq$25-<50<br>○ Insufficiency: $\geq$50-<75<br>○ Sufficiency: $\geq$75<br>• Statistical comparisons of vitamin D level/status were made among the different groups.	• The ASD group had significantly lower levels of 25(OH)D_3 compared to the control, and to their siblings and parents.<br>• In the ASD group, 88% were vitamin D deficient (specifically, 53% severe deficiency). Among their siblings, parents, and the control group, the corresponding rates were 58% (16%), 59% (23%), and 65% (23%), respectively ($p<0.001$).<br>• There was no association between 25(OH)D_3 levels and age, intellectual quotient, subcategories of ASD, and gender.<br>• As all groups were exposed to low levels of sunlight necessary for vitamin D biosynthesis, the very low serum

Reference	Aim	Study Design & Sample	Methodology for outcomes of interest	Results & Conclusions
		o 77 typically developing parents: 52% females<br>o 40 healthy age- and gender-matched controls		levels in the ASD group could be either a result of autism impacting on diet or the underlying biology of autism altering the metabolism of vitamin D in some way.
**Clinical assessment**				
Croen *et al.,* 2015 [7] USA	The objective was to describe the frequency of medical and psychiatric conditions among a large, diverse, insured population of adults with autism in the US.	• Cross-sectional, case-control study<br>• 1,507 adults with ASD formally diagnosed:<br>o 27% female<br>o 29.0+12.2 (from 18 to >65) years old<br>o White 70%; Black 8%; Asian 11%; Other 11%<br>o At least 19.2% ID<br>• 15,070 age- and sex-matched typical controls.	• Prevalence of health conditions was calculated from data documented in electronic medical records.<br>• Prevalence of vitamin deficiency was compared between groups using chi-square tests. A multivariate logistic regression model was run to compare the OR of the condition between ASD cases and controls after controlling for sex, age, and race/ethnicity.	• Vitamin deficiency was diagnosed more frequently among autistic females compared to males (9.6% vs. 3.3%), although the prevalence is low in both cases.<br>• Adults with ASD had significantly higher prevalence of vitamin deficiency than controls [5% vs. 2.3%; OR (99% CI) = 2.35 (1.65-3.33)]<br>• Both men [OR (99% CI) = 2.24 (1.36-3.68)] and women [OR (99% CI) = 2.46 (1.50-4.04)] had increased risk compared to unaffected controls.
**Dietary assessment**				
Blomqvist *et al.,* 2015 [57] Sweden	The hypothesis to be tested were that adults with ASD have a higher caries prevalence, have more risk factors for caries development, and utilize dental health care to a lesser extent than people recruited from the normal population.	• Cross-sectional, case-control study<br>• 47 adults with ASD formally diagnosed:<br>o 47% female<br>o 33+8 years old<br>o Without ID, but a wide range of severity (AQ from 5 to 46)<br>• 69 age- and sex-matched typical controls.	• Each participant attended a dental examination appointment and completed questionnaires on Demographic & background information, Oral health & dental hygiene habits, and Dietary habits<br>• Frequencies of the dietary habits that are most predictive of caries risk were calculated and comparisons between groups were performed.	• The ASD group had less snacking i.e. eat/drink any food/beverage >5 times/day (51% vs 71%, p=0.029).<br>• Both groups reported similar habits regarding:<br>o Eat mints, hard/chewy candies, candy bars, donuts, pastries, chips, crackers or other similar snack foods between meals 3 days/week (38% vs 43%, p=0.578).<br>o Drink non-diet soda, lemonade, fruit aids, sport drinks, or sugar (not-sugar substitute)-sweetened tea or coffee between meals (40% vs 46%, p=0.526).<br>• The comparison between the two groups showed no differences in terms of the consumption of potentially cariogenic foods or even a lower snacking habit for the ASD group. In any case, the frequency of consumption of high-caloric low-nutrient dense foods was high.
Nakamura *et al.,* 2019 [58] Japan	The study aimed to investigate an association between dietary intake and autistic traits.	• Cross-sectional, observational study. Sub-study of the *Eating Habit and Well-Being* study of Japanese manufacturing workers	• Validated 87-food item FFQ developed for Japanese people.<br>• Information on dietary and nutrient intake stratified by sex was analyzed. The	• Autistic traits were associated with low intakes of several nutrients, even in non-clinical Japanese adults from the general working population. These low intakes may be related to lower consumption of several food items:

Reference	Aim	Study Design & Sample	Methodology for outcomes of interest	Results & Conclusions
		• 2,053 adults, 30% females, with some level of autistic traits assessed using the *Japanese version of the Subthreshold Autism Trait Questionnaire, SATQ*.<br>o Male: 20.3% high and 49.5% moderate SATQ score<br>o Female: 13.5% high and 43.6% moderate SATQ score	association of the SATQ score with the nutrient or food intake was examined using linear regression analyses	o Iron and vitamin B12 intakes were marginally and inversely associated with the SATQ score in men.<br>o SATQ score in women was positively associated with carbohydrate intake, but had an inverse association with protein, fats, mineral, vitamin, and dietary fiber intakes.<br>o Intakes of seaweed, and fish and shellfish had an inverse association with the SATQ score in men.<br>o The SATQ score in women was positively associated with grain products intake, but had an inverse association with the vegetables, mushrooms, and fruit intake.<br>• Association between nutrient/dietary intake in women was more evident than that in men.
Weir *et al.*, 2021 [53] Several countries, mostly UK (71%), USA (10%)	This study examines whether obesity-related dietary, exercise, and sleep patterns are seen among autistic adults, as well as whether these lifestyle factors contribute to the elevated risks of chronic diseases seen among autistic adults.	• Cross-sectional, case-control study<br>• 1,183 participants with ASD formally diagnosed:<br>o 63% female<br>o 41.0+14.4 years old (range 16 to 90).<br>o White 88%; Multiracial 6%; Other 6%.<br>o Moderate to severe ID excluded; 1.8% ID self-identified.<br>o 59% university studies, 18% secondary/high school, and 23% lower level of studies<br>• 1,203 age-matched typical controls	• Information about participants' dietary habits was collected through an online survey (https://www.qualtrics.com)<br>• Due to sociodemographic differences between ASD and control groups, adjusted models (logistic regression) were applied to assess diet patterns.	• Autistic adults were more likely than non-autistic to have almost of all the tested dietary restriction or preference (vegan, vegetarian, lactose-free, gluten-free, no fish, and other) as well nearly to a double of autistic adults reported dietary restriction due to allergy.<br>• They were also more likely to eat foods high in calories, fat, salt, or sugars (for example: fried foods, chocolate, cakes, ice cream, etc) frequently (i.e. $\geq$ 7 times per week)<br>• Autistic females, but no males, were less likely to eat 5 servings of F&V on at least 4 days per week than sex-matched peers.<br>• They were found marginally statistically significant differences in the likelihood of meeting daily water goals (i.e $\geq$ 8 cups/glasses), or drinking high sugar beverages (soft drinks, fruit juice, smoothies, etc.) frequently, but autistic adults reported drinking less caffeinated beverages (tea, coffee, energy drinks, etc)
Kranz *et al.,* 2022 [59] USA	The aim of this explorative pilot study was to examine parents' perception of food intake for themselves and their young adult children with ASD and explore the potential for perceived intergenerational transfer of	• Cross-sectional population-based study<br>• 488 parents (or primary caregivers) of young adult children with ASD recruited from "Autism Speaks'" database & an internet search of schools/facilities that served them.	• Dietary patterns data was collected through internet-based completion of a survey instrument (https://www.qualtrics.com) using parent's self-reported responses.<br>• Questionnaire had 14 questions regarding dietary intake about:<br>o Information on several factors of dietary intake patterns	• Most respondents (76.8%) reported their children consumed a varied diet across food groups, which aligned with the finding that 66.8% avoided eating the same foods daily.<br>• Snacking was prevalent, with 75.8% of participants either regularly or occasionally consuming between-meal snacks, predominantly preferring sweet (44.4%) and salty (26.2%) options over fruits and vegetables (7.4%).

Reference	Aim	Study Design & Sample	Methodology for outcomes of interest	Results & Conclusions
	dietary intake patterns between them.	● Characteristics of the young adult children:<br>o ASD (diagnosed by physician or school psychologist):<br>- Level 1: 47.6%<br>- Level 2: 21.7%<br>- Level 3: 11.4%<br>- Missing/not specified: 19.4%<br>o 25.3% female (Missing/not specified: 25.5%)<br>o 21.8+3.7 years old (range 18 to 28)<br>o Under care in same residence as the parent (or primary caregiver).	o Consumption of commonly under-consumed foods in individuals with ASD.	● Regarding hydration, approximately half (49%) meet the recommended daily water intake of eight glasses, with a preference for cold beverages (39.8%) over hot drinks (12.9%).<br>● Nutritional supplementation is common, with 50.4% of respondents taking supplements, primarily in the form of multivitamin and mineral combinations (38.9%).<br>● 52% reported their children were picky eaters in early childhood, but 48.2% now enjoy previously disliked foods, suggesting significant evolution in food preferences.<br>● Current dietary patterns show diverse preferences across food groups. In vegetables, white and starchy varieties (22.1%) and orange vegetables (21.7%) are most frequently consumed. Protein choices are led by red meat (29.4%) and poultry (27.2%), while grain consumption is evenly distributed between white bread (24.6%) and whole grain options (21.8%). For fats and oils, olive oil and other vegetable oils (31.1%) are most used, followed by butter (24.8%).
Docherty, 2023<br>[60]<br>USA	The purpose of this research was to develop and implement a curriculum educating adults with ASD on meal planning and preparation to increase nutrition and cooking knowledge and skills, and readiness to change.	● Educational intervention study without control group.<br>● 7 university students with ASD enrolled in the Summer LIFE @ the Beach and LIFE Project programs at California State University, Long Beach:<br>o 29% female<br>o 21.0±3.6 (range 18 to 29) years old<br>o White 43%; Hispanic 29%; Other 28%<br>o 100% living with parents or guardians	● Pre-test survey questions included sociodemographic information, and last month's dietary intake (fruits, vegetables, potatoes, and dried beans) and dietary restrictions as part of the sample description who participate in the educational intervention. Dietary intake questions were adapted from fruit and vegetable module of *Behavioral Risk Factor Surveillance System (BRFSS)* survey by the *Centers for Disease Control and Prevention (CDC)*<br>● Mean frequencies of the different responses were reported.	● None of participants reported (religious or health-related) dietary food restrictions<br>● Food consumption:<br>o Fruits: Almost 5-6 times per week fresh, canned or frozen fruit. Less than 1-2 times per week 100% fruit juice, and mixtures than included fruit.<br>o Potatoes: More than 1-2 times per week French fries or potato chips, an almost 1-2 times per week other white potatoes (baked, boiled, mashed or any other preparation that was not fried)<br>o Vegetables: Almost 3-4 times per week mixtures that included vegetables (sandwiches, casseroles, soups, stir-fry, omelets, and tacos), and less than 1-2 times per week lettuce salad. Less than 4 times last month "all other vegetables" (raw, cooked, canned, and frozen)<br>o Less than 1-3 times last month cooked dried beans (chili, baked beans, bean soup, and other bean dishes)

Reference	Aim	Study Design & Sample	Methodology for outcomes of interest	Results & Conclusions
Garcia *et al.,* 2023<br>[61]<br>USA	The aim was to develop and evaluate participant acceptability and the feasibility of recruitment, retention, adherence, and implementation of a nutrition education and culinary skills intervention for young adults with ASD.	• Educational intervention study without control group.<br>• 13 participants with level I ASD diagnose by a physician, affiliated with a community organization that provides services to individuals with ASD and their families in the Central Florida area:<br>o 23% female<br>o 26.2±4.5 years old<br>o White 77%<br>o 31% currently employed<br>o 85% lived with family<br>o 77% with additional diagnosed health condition (ADHD; anxiety disorder; mood disorder), 54% currently taking medication, 16% known food allergies	• An online survey (https://www.qualtrics.com) included several questions on sociodemographic and clinical data, and 2 questions on diet (quality & habits) as part of the sample description who participate in the educational intervention<br>• Frequencies of the different responses to diet quality questions were calculated.	• *Do you define your current diet as healthy?*<br>o Yes: 8%<br>o Sort of healthy: 54%<br>o No: 38%<br>• *How many times do you consume fast-food or take out per week?*<br>o Every day: 0%<br>o 3-5 times per week: 16%<br>o 1-2 times per week: 38%<br>o 1-2 times per month: 23%<br>o Never eat: 0%<br>o Not sure/it varies: 23%

ASD = Autism Spectrum Disorder; ID = Intellectual Disability; OR (99% CI); Odds Ratio (99% Confidence Interval); AQ = Autism-Spectrum Quotient; SATQ = Japanese version of the Subthreshold Autism Trait Questionnaire; ADHD = Attention Deficit Hyperactivity Disorder

Table S4: **Nutritional supplementation and restricted diets as therapeutic strategies in adults with ASD**

Reference	Aim	Study Design & Sample	Methodology for outcomes of interest	Results & Conclusions
**Evaluation of nutritional supplementation**				
**Adams** ***at al.*****, 2022** [62] USA	To evaluate the safety and efficacy of ANRC-Essentials Plus (ANRC-EP), a vitamin/mineral/micronutrient supplement, in children and adults with autism spectrum disorder (ASD)	• Participants: o 161 participants with a formal diagnosis of ASD o 17 participants over 21 years and 31 (age 16-20) o Autism severity: Mild 39 (24%), Moderate 76 (47%), Severe 45 (28%) • Design: Retrospective survey of consumers who purchased ANRC-EP for a child or adult with ASD. • Inclusion criteria: Formal diagnosis of ASD and use of ANRC-EP for 3 months or longer.	• Survey: An email invitation to complete a ten-minute survey about the safety and efficacy of ANRC-EP. • Evaluations: Parent Global Impressions of Autism (PGIA) and the Overall Benefit/Adverse Effect scale of the National Survey on Treatment Effectiveness for Autism (NSTEA) • Results were compared with a three-month randomized double-blind placebo-controlled study of an earlier version of the supplement (Adams 2018)	• PGIA Scores: Participants reported substantially higher Average PGIA Scores than the placebo group in a similar previous study, with an estimated effect size of 0.66. • Overall Benefit: 73% of participants rated the Overall Benefit as Moderate, Good, or Great. • Adverse Effects: The Overall Adverse Effect score was low (0.25/3.0). • Sub-analysis: The Overall Benefit of ANRC-EP was not significantly affected by gender, age, autism severity, diet quality, self-limited diet, use of psychiatric or seizure medications, dosage, developmental history, intellectual disability, or seizures.
**Lundbergh,** ***et al.*****, 2022** [63] DENMARK	To explore the effects of n-3 long-chain polyunsaturated fatty acids (n-3 LCPUFA) on cognitive functions in adults with autism spectrum disorder (ASD) and to determine if these effects are modified by comorbid	• Participants: 26 adults aged 18-40 with a self-reported clinical diagnosis of ASD • Design: 2 × 4 week randomised double-blind crossover trial with fish oil (FO) and safflower oil (SO) supplementation.	• Participants were tested at baseline and at the end of each intervention period o Primary outcomes: Attention (d2-test) and spatial working memory (Corsi test). o Secondary outcomes: Flexibility (Stroop test), ADHD symptoms (Conners scales), executive functions (BRIEF-A), and social behaviour (SRS-2).	• Improvement in Corsi scores and reduction in d2 test errors after FO compared to SO. • Improved Conners scores of attention, particularly in participants with ADHD. • Participants without ADHD showed the most benefit in d2 test performance but had exacerbated executive function scores after FO.

Reference	Aim	Study Design & Sample	Methodology for outcomes of interest	Results & Conclusions
	attention-deficit/hyperactivity disorder (ADHD).	• Two 1-month periods of supplementation with FO and SO, respectively, without a washout period.<br>• Randomisation: Participants were randomised to either FO → SO or SO → FO sequence	o Compliance: Assessed through self-report, capsule counts, and whole-blood EPA + DHA analysis.<br>• Background information about diagnoses and medication, as well as living conditions and lifestyle, was collected in an interview at the baseline visit.	• No significant effects on core ASD symptoms.<br>• FO supplementation may improve attention and working memory in adults with ASD, and FO may ameliorate ADHD symptoms in those with comorbid ADHD.
**Geng *et al.*, 2021** [64] USA	To gather opinions on the effectiveness of specific food blends and nutrients on speech and motor impairments in individuals with communication delays and/or motor dysfunction symptoms	• Participants: 77 individuals (age 2 - 70 years), not specify number of adults<br>• Design: A 28-point web-based survey targeting consumers of IQed nutritional (food blend product,)	• Survey Instrument: Designed to capture data on symptoms of speech and motor abilities, including multiple-choice, rating scale, and open-ended questions.<br>• Recruitment: Participants were recruited from a list of past consumers who had used the product for more than one month.	• 92% of respondents reported positive changes in behavior or physical symptoms.<br>• Improvements were noted in expressive speech (85.7%), vocalizations (88.1%), speech (77.6%), oral motor skills (63.2%), receptive ability (69.6%), focus (65.1%), motor planning (77.6%), mood (62.3%), social skills (59.3%), and physical/behavioral health (47.3%).
**Evaluation of nutritional supplementation and restricted diets**				
**Adams *et al.*, 2018** [65] USA	The goal of this study is to investigate a comprehensive nutritional and dietary intervention to treat children and adults with ASD	• Participants:<br>○ 67 children and adults with ASD (ages 3-58 years) and 50 neurotypical controls.<br>○ Adults: ASD treatment group: 6 teenagers (age 13-20) and 3 adults (age more than 20), ASD non treatment group: 7 teenagers (age 13-20) and 3 adults (age more than 20), non-sibling neurotypical controls: 11 teenagers (age 13-20)/ 5 adults (age more than 20).<br>• Design: Randomized, controlled, single-blind 12-month study<br>• Groups: ASD treatment group, ASD non-treatment group, and neurotypical control group.	• Initial evaluation of autism severity and overall functioning.<br>• Sequential addition of treatments: vitamin/mineral supplements, essential fatty acids, Epsom salt baths, carnitine, digestive enzymes, and a healthy gluten-free, casein-free, soy-free (HGCSF) diet.<br>• Biomarker measurements in blood and urine at the beginning and end of the study.<br>• Assessments of autism severity and overall functioning: Parent Global Impression (PGI-2), Autism Diagnostic Observation Schedule (ADOS), Reynolds Intellectual Assessment Scales (RIAS), Childhood Autism Rating Scale 2 (CARS-2), Severity of Autism Scale (SAS-Pro), Vineland Adaptive Behavior Scale II (VABS-II)	• Significant improvement in non-verbal IQ in the treatment group (+6.7 ± 11 IQ points vs. ?0.6 ± 11 IQ points, p = 0.009).<br>• Greater improvement in autism symptoms and developmental age in the treatment group.<br>• Significant increases in EPA, DHA, carnitine, and vitamins A, B2, B5, B6, B12, folic acid, and Coenzyme Q10 in the treatment group.<br>• Parents reported the vitamin/mineral supplements, essential fatty acids, and HGCSF diet as the most beneficial treatments.
Evaluation of restricted diets as therapeutic strategies				
**Matthews & Adams, 2023** [66]	The study was designed to obtain an understanding of the benefits and adverse effects of	• Participants: 818 participants and 25% were over 18 years old (202 adults)	• Evaluation of casein-free diet, corn-free diet, Feingold Diet (defined as no artificial colors, flavors, or preservatives), food-avoidance diet (based on IgG or IgE	• Top-rated diets by Overall Benefit: Healthy diet (2.7), Feingold diet (2.6), and food avoidance diet based on IgG/IgE testing (2.6).

Reference	Aim	Study Design & Sample	Methodology for outcomes of interest	Results & Conclusions
USA	therapeutic diets for individuals with autism spectrum disorder, as rated by caregivers of children and adults with ASD (and some individuals with ASD)	• Design: Cross-sectional observational study using an online survey.<br>• The inclusion criteria were parents and caregivers of children and adults with ASD, as well as individuals with autism spectrum disorder. Since participation was anonymous, diagnosis was not verified.	food testing), food-avoidance diet (based on observation), GAPS (Gut and Psychology Syndrome) Diet, gluten-free and casein-free (GFCF) diet, gluten-free diet, healthy diet (defined as high intake of vegetables, fruit, protein; low intake of junk food), ketogenic diet, low oxalate diet, low sugar diet, medium chain triglyceride diet, modified Atkins diet, Paleo diet, rotation diet, soy-free diet, and Specific Carbohydrate Diet (SCD) and none.<br>• Survey included questions on medical history, psychiatric and seizure medication, general medication, nutraceuticals, diets, therapies, and education. Participants rated the Overall Benefit (0-4 scale) and Overall Adverse Effects (0-3 scale) of each diet.	• Ketogenic diet was highest for nine symptoms, including attention, cognition, and anxiety.<br>• GFCF diet was among the top for overall symptom improvements.<br>• Significant improvement in autism severity for diet users compared to non-diet users ($p < 0.001$).<br>• Therapeutic diets can be safe and effective interventions for improving some ASD-related symptoms with few adverse effects.<br>• Different diets affect different symptoms, suggesting a personalized approach based on individual symptoms.

ASD = Autism Spectrum Disorder

Table S5. **Dietary intervention to implement a healthy diet for adults with ASD**

Reference	Aim	Study Design & Sample	Methodology for outcomes of interest	Results & Conclusions
**Conti *et al.,* 2024**<br>[67]<br>ITALY	To develop canteen menus that meet the nutritional and sensory needs of adults with Autism Spectrum Disorder (ASD) to reduce food selectivity and improve their health	• Design:<br>o Intervention pilot study<br>o 3 months' study conducted at Italian daycare service<br>• Sample:<br>o Twenty-two individuals with ASD, aged 19–48, 72.7% males, were enrolled.<br>o Overweight and obesity prevalence were 54.5 and 18.2%, respectively.	• Two main phases:<br>o WP 1 Observational phase: a comparison was made between the enrolled subjects' nutritional needs and the nutrient content of the administered menus during the daycare service. Then mealtime compliance was assessed using standardized meal evaluation forms, both quantitative and qualitative<br>o WP2 Intervention phase: canteen menus targeted to the individuals' nutritional and sensory needs were administered and their acceptability was evaluated	• Identified that the most accepted foods had specific sensory characteristics such as low-intensity colors, soft textures, and faint smells.<br>• The results describe an increase in the score of aceptability for all the meal dishes (first course, second course, vegetable side dish, fruit) at WP2 compared to WP1.<br>• The results highlighted the need for adapted menus and greater attention to the way meals are delivered and consumed to improve nutritional status and therefore health of this population at increased risk of malnutrition.
**Veneruso et al., 2022**<br>[68]<br>ITALY	The Il Tortellante® is an Italian project aimed at promoting adaptive behavior and social skills, and at reducing the severity of symptomatology through a culinary	• Design: A pre-post design study was performed.<br>• Sample:<br>o 20 patients aged between 15 and 25 years were recruited, diagnosed with ASD<br>o The mean age of participants was 19.3 years ± 3.58	• Data were collected at the start of the project in September 2018 (pre-test) and after the intervention in April 2021 (post-test). Activities were discontinued from March 2019 to May 2020 due to the SARS-CoV-2 pandemic<br>• The severity of symptoms (The Childhood Autism Rating Scale, Second Edition (CARS-2), social skills (Social Responsiveness Scale (SRS)),	• CARS 2 scores improved significantly from 35.07 to 32.42 ($p = 0.007$), with symptom severity reduced in four patients. While SRS scores showed no significant change ($p = 0.409$), two patients improved from severe to mild social impairment. Vineland II Composite scores remained low, but Daily Living Skills improved significantly ($p = 0.041$). Positive trends were observed in

Reference	Aim	Study Design & Sample	Methodology for outcomes of interest	Results & Conclusions
	group intervention in which young people with ASD learn to make fresh pasta by hand.		and adaptive behaviours (Vineland Adaptive Behavior Scale II (VABS II) were assessed before and after the intervention<br>● Patients attended supervised training to learn fresh pasta production and social skills, with sessions tailored to individual needs using TEACCH and ABA methods. The program included personalized education, group activities, and projects like pantry management, money handling, social skills, emotional awareness, and independent living, aiming to enhance autonomy and reduce ASD-related symptoms.	social cognition, though most domains showed no significant changes.<br>● Regression of the symptom severity and improvement in daily living skills suggests that a culinary laboratory that combines work and social participation could be an interesting approach to improve the quality of life of ASD patients
Nabors *et al.*, 2021 [69] USA	The pilot study aimed to evaluate the structure, implementation, and outcomes of a healthy eating and exercise program for these young adults.	● **Design:** Pilot study<br>● Sample: The study involved 17 young adults with Autism Spectrum Disorder (ASD) and intellectual disabilities (IDs), six parents, and 10 staff members.	● Participants attended weekly lessons on healthy eating, exercise, and related health topics from January 2020 to April 2021.<br>● The program initially used the Traffic Light Diet but switched to MyPlate and other visual aids after feedback.<br>● Lessons were delivered in small groups, both in-person and online during the COVID-19 pandemic.<br>● Goals were set using group-based motivational interviewing.<br>● Data were collected through height and weight measurements, parent surveys, and group interviews with participants.	● Two participants lost significant weight, and others maintained their weight during the pandemic.<br>● Participants and parents reported increased knowledge and behavior changes regarding healthy eating and exercise.<br>● The program was well-received, with positive feedback from both participants and parents.<br>● The study showed promising results for using group-based motivational interviewing to promote healthy behaviors in young adults with ASD and IDs.
Gustin *et al.*, 2020 [70] USA	The study assessed the impact of a six-week cooking course on their cooking skills, frequency of self-prepared meals, and confidence in meal preparation.	● Design: Pilot study<br>● Sample: The study involved 11 college students with autism spectrum disorder (ASD) enrolled in the Learning Independence for Empowerment (LIFE) Project at a large urban state school in Southern California.	● The cooking course was designed and taught by registered dietitian nutritionists and included six cooking modules.<br>● The curriculum featured visual recipe guides and sensory-friendly recipes.<br>● Pre-and post-tests were administered to measure changes in cooking methods, frequency of self-prepared meals, and confidence in cooking ability.<br>● Data were collected over two years.	● The results showed significant improvements in the frequency of meals prepared by students and their confidence in cooking ability.<br>● The frequency of meals prepared from basic ingredients increased by about one meal per week.<br>● Confidence in cooking from basic ingredients and tasting new foods also showed substantial increases<br>● The study demonstrated that a hands-on cooking course could enhance cooking skills and confidence among young adults with ASD, promoting independence and better dietary habits.

Reference	Aim	Study Design & Sample	Methodology for outcomes of interest	Results & Conclusions
Hubbard *et al.*, 2015 [71] USA	The study evaluated a Smarter Lunchroom intervention over three months at a residential school for students aged 11–22 with intellectual and developmental disabilities (I/DD).	• Design: Pilot Intervention study (The quasi-experimental, pre-post design compared five days of dietary data before and after the intervention).<br>• Sample: 120 students aged 9–22 years. not specify number of adults	• The intervention used behavioral economics principles to nudge students towards healthier food choices by modifying the dining environment.<br>• Changes included improved food visibility, repositioning healthier options, and unbundling side dishes. Data on food selection and consumption were collected using digital photography.	• The intervention significantly increased the selection and consumption of whole grains and fruits while reducing refined grains and fruit/vegetable plate waste.<br>• However, there was no change in total energy intake. Positive trends suggested improved dietary composition without adverse behavioral effects.

ASD = Autism Spectrum Disorder
